# Partner support and goal outcomes during COVID‐19: A mixed methods study

**DOI:** 10.1002/ejsp.2745

**Published:** 2021-05-05

**Authors:** Laura M. Vowels, Katherine B. Carnelley, Rachel R. R. Francois‐Walcott

**Affiliations:** ^1^ School of Psychology University of Southampton Southampton UK

**Keywords:** COVID‐19, goal outcomes, goals, interpersonal relationships, partner support

## Abstract

During the COVID‐19 pandemic, people have been stuck indoors with their partners for months. Having a supportive partner is likely to be especially important during this time when access to outside sources of support is limited. The present mixed‐methods study aimed to investigate how partner support is associated with goal outcomes during COVID‐19. The survey participants (*n* = 200) completed a daily diary for a week and five weekly longitudinal reports, and 48 participants attended a semi‐structured interview. The quantitative results showed that higher relational catalyst support (i.e., support for growth opportunities) predicted better goal outcomes; qualitative analyses revealed partners use direct and indirect forms of emotional and instrumental support toward goal pursuit. This is important because most studies to date have not differentiated between direct and indirect forms of support. Overall, the findings suggest that having a supportive partner is important for not only surviving, but also thriving through the pandemic.

## 1 INTRODUCTION

The world is currently experiencing unprecedented times that are changing the nature of society as we know it. The COVID‐19 outbreak has led many countries to implement social distancing measures such as working from home, avoiding social contact, and closing schools that have social and economic implications (United Nations, 2020). As such, close relationships have been uniquely impacted with more couples staying indoors for prolonged periods of time to take care of children and the household, as well as completing work tasks (Carlson et al., 2020). Partner support is especially crucial during the pandemic because one's partner may be the only person available for support toward tasks and goals, while at the same time partners may be preoccupied with the demands caused by the pandemic. In the present mixed‐methods study, our aim was to understand how partner support may have been affected during the pandemic and whether perceiving one's partner as supportive is associated with better goal outcomes during the pandemic. We examined the association between partner support and goal outcomes in the daily diary and longitudinal quantitative surveys. In qualitative interviews, we asked participants how partners were supporting each other and in what ways the support had changed since the pandemic started to provide a more nuanced understanding of the impact of the pandemic on support.

A recent theoretical model, thriving through relationships, describes the interpersonal process of how partners can create an optimal environment for goal outcomes by providing Relational Catalyst (RC) or Source of Strength (SOS) support (Feeney & Collins, 2015). RC support is an extension upon attachment theory's (Bowlby, 1969) notion of a *secure base* and “functions to promote thriving through full participation in life opportunities for exploration, growth, and development in the absence of adversity” (Feeney & Collins, 2015, p. 118). SOS support, in turn, is an extension of a *safe haven* and “functions to promote thriving through adversity, not only by buffering the negative effects of stress but also by helping others to emerge from the stressor in ways that enable them to flourish” (Feeney & Collins, 2015, p. 118). In essence, SOS support is similar to dyadic coping (Bodenmann, 1997; Falconier et al., 2015; Falconier & Kuhn, 2019), which is often conceptualized as the way in which partners help each other cope in stressful situations. Therefore, both SOS support and dyadic coping are focused on coping with the stressful situation itself whereas RC support is more concerned with pursuing opportunities and supporting exploration and growth. While both types of support are likely to be important during the pandemic, the present study focused on RC support. We argue that while the pandemic is an ongoing, unpredictable situation, most people have to continue to pursue goals and tasks despite the pandemic. Therefore, because outside support is likely to be limited during this time, the extent to which partners provide RC support is likely to be especially important in order for individuals to continue to make progress toward their goals.

RC support is provided through partners being an active catalyst during the process of achieving goals and includes four components: (a) nurturing opportunities for growth by providing encouragement, validating goals, and expressing enthusiasm for new opportunities; (b) providing perceptual assistance in recognizing and perceiving opportunities as challenges rather than threats, (c) providing practical assistance in the preparation of pursuing life's opportunities, and (d) serving as a launching function to help one's partner fully engage in life's opportunities by providing a secure base for exploration, celebrating successes, and assisting in dealing with adjustments or setbacks (Feeney & Collins, 2015). If the partner is able to provide effective RC support, the recipient is likely to perceive the partner as responsive, which leads to immediate as well as long‐term thriving outcomes (Feeney et al., 2017; Tomlinson et al., 2016). In contrast, if a partner provides anti‐RC support (i.e., support that is intrusive or unwanted), the recipient is likely to experience lower thriving outcomes. To date, there are no studies that have examined whether self‐reported perception of RC support from partners predicts goal outcomes as previous studies have relied on observers' perceptions only.

However, there are other studies that have been conducted over the past three decades that have examined the association between partner support on goal outcomes. For example, several studies have noted that perceiving one's partner as supportive is associated with greater goal progress (Brunstein et al., 1996; Drigotas et al., 1999; Feeney, 2004; Jakubiak & Feeney, 2016; Kumashiro et al., 2007), commitment toward goals (Dailey, 2018; Feeney, 2004; Low et al., 2017; Overall & Fletcher, 2010), and confidence in one's abilities to achieve goals (Feeney, 2004; Low et al., 2017; Tomlinson et al., 2016; Winterheld & Simpson, 2016). Although fewer studies have examined negative support, some have found that negative support is associated with lower goal confidence (Feeney et al., 2017; Hammond & Overall, 2015) but not with goal progress or commitment (Overall et al., 2010). While the majority of the studies show that support is beneficial for goal outcomes, there are other studies that have found that support can at times be costly as it may hinder self‐efficacy (Bolger et al., 2000; Crockett et al., 2017; Girme et al., 2013; Gleason et al., 2008). Overall, though, a recent meta‐analysis found that partner support was moderately positively associated with goal outcomes (Vowels et al., 2021).

All of the aforementioned studies have been conducted either in non‐stressful situations or in situations in which only one member of the dyad experienced the stressor (professional stressor [Bolger et al., 2000]; and a laboratory stressor [Crockett et al., 2017; Gleason et al., 2008]) and therefore, the non‐stressed partner may have been more available to provide support. During the COVID‐19 pandemic, both partners are experiencing the stressor simultaneously and the stress is likely to be prolonged with uncertainty around when the stress may be alleviated. The prior research examining the association between partner support and goal outcomes in stressful times has focused on one partner's stress with a specific end‐date or a single induction of stress. We expect that in line with the majority of the previous research, RC support will be positively, and anti‐RC support negatively, associated with goal outcomes (progress, confidence, commitment; H1).

The coronavirus pandemic may create a need for more support between couples as they manage pandemic‐induced stress alongside the pursuit of tasks and goals (e.g., work, education, health, domestic). Nonetheless, these exceedingly stressful times may leave couples unable to respond sensitively to their partners' needs (Neff & Karney, 2004). Early research into the impact of COVID‐19 on relationships found that COVID‐19 related stressors (financial strain, stress, and social isolation) negatively impacted relationship quality and conflict, but perceiving partner as responsive buffered against the negative impact of the stressors (Balzarini et al., 2020). Therefore, it may also be that partner support can buffer against potential negative impacts of the pandemic on goal pursuit. Although there are no studies to date that have addressed this question, we expect that perceiving partner as more supportive will be associated with perceiving the pandemic as affecting goal pursuit less negatively (H2).

Furthermore, our aim is to add to the current understanding of what types of support partners provide during COVID‐19 and how support may have been changed as a result of the pandemic (RQ1). While the thriving through relationships framework (Feeney & Collins, 2015) proposes that in stressful times, partners' primary role is to provide support that offers relief from stress (SOS support), the framework suggests that RC support is provided in the absence of adversity. However, we argue that RC support can also be effective in times of adversity, such as during the COVID‐19 pandemic, to enable pursuit of tasks and goals that may still need to be completed (e.g., work tasks, children's homeschooling, exercise). During these times, the goals may be qualitatively different compared to non‐stressful times in that they may focus more on immediate tasks rather than long‐term goals but partner support may still be needed in order to accomplish them. Qualitative studies are especially useful in understanding what types of support individuals may be providing during the pandemic. Therefore, our hope is to extend the thriving through relationships framework by exploring the ways in which RC support can still be effectively provided in times of stress. Understanding how partners can support each other to pursue tasks and goals during the pandemic can help partners and the relationship not only to survive through the pandemic, but to bounce back and thrive beyond the pandemic.

## 2 METHOD

### 2.1 Participants and procedure

We preregistered the study on the Open Science Framework, which can be found here: https://osf.io/esa3u/?view_only=a1324d6d57ed4d5b80a024e5d80b0e19. Data, code, and materials can be found here: https://osf.io/qr7cm/?view_only=365bf35f7ddd45548143b851e10cfcd9. Ethical approval was received from the authors' institutional review board. We collected the quantitative data via Prolific and used random sampling via social media to recruit participants for the qualitative interviews. Participants were eligible for the study if they were 18 years old or above and living with their romantic partner in a country where social distancing measures were in place. The participants were informed that the study focused on “understanding how the coronavirus pandemic is affecting people's day‐to‐day lives and relationships while living in close quarters with their partners/families for an extended period of time”. Due to funding, the number of participants for the quantitative surveys was constrained to 200. Based on a simulated power analysis, data from 200 participants (4,200 observations in the daily diaries) yield a power of 96.7% to estimate an average effect size in Psychology (*r* = .22, *d* = 0.45; Richard et al., 2003) with an alpha level of *p* < .01 and an estimated intra‐class correlation of .30. Participants recruited through Prolific received £4.70 for the daily diary and an additional £2.00 after all follow‐ups were completed. Qualitative interview participants were entered into a raffle to win one of two £30 Amazon vouchers after the first interview and one of two £20 Amazon vouchers after the second interview.

**FIGURE 1:**
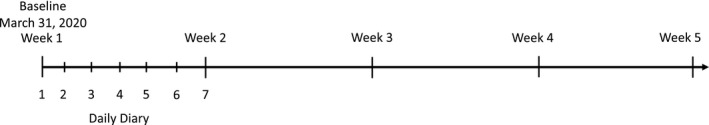
A graphical illustration of the study timeline

All survey participants completed a baseline survey on March 31, 2020, shortly after many countries had gone into lockdown. Participants then completed a daily diary survey over the next seven days with the first entry completed directly after the baseline survey. After the daily diary portion of the study, participants completed a further three follow‐up surveys that were each one week apart. This resulted in a total of five weekly time‐points (see Figure 1 for study timeline). Participants responded to questions regarding partner support and goal outcomes from the previous 24 hours in the daily diaries and from the previous week in the follow‐up surveys. All surveys were conducted via Qualtrics. The final sample in the quantitative surveys was 200 with an attrition rate of 4% at the end of the daily diary and 8.5% at the end of the five weeks. However, all participants completed at least two time‐points and were therefore included in the final analyses.

The semi‐structured qualitative interviews were conducted via Zoom, audio recorded, and transcribed. All first interviews were completed between March 30, 2020 and April 21, 2020. A total of 48 participants completed the first qualitative interview (30 were recruited via social media, 18 via Prolific who participated in both quantitative and qualitative parts of the study). We invited participants who had completed the first interview in the first two weeks of the qualitative data collection to participate in the follow‐up interview to better understand how support had changed over the course of the lockdown.^1^ Nineteen of the 23 participants invited to complete a second interview responded. The initial interviews lasted between 14 and 49 min and second interviews between 7 and 24 min.

Participants in quantitative and qualitative portions of the study had similar demographic characteristics (see Table 1). Participants were 36 years old on average and had been in a relationship for 11 years. The samples were primarily white, heterosexual, and from the UK. Around half the participants were married and half cohabiting, and half of them had children. Only a small number of participants were keyworkers^2^ or had shown coronavirus symptoms. None had been diagnosed with coronavirus at baseline. Participants had been under social distancing rules between seven to 42 days (*M* = 10.85, *SD* = 6.94). At baseline, all but two (living in the US, working from home) of the participants were under partial (only going out if absolutely necessary) or full lockdown (not leaving the house). On average, participants reported low positive mood (*M* = 3.07, *SD* = 4.75 on a scale between −10 to 10) with mild to moderate levels of depression (*M* = 3.31, *SD* = 2.29 on a scale between 0 to 10) and anxiety (*M* = 3.56, *SD* = 2.44 on a scale between 0 to 10).

**TABLE 1:** Demographic variables for the quantitative and qualitative data

	Quantitative (*n* = 200)		Qualitative (*n* = 48)^a^	
	*M*	*SD*	*m*	*SD*
Age	36.5	12.3	36.0	12.9
Relationship length	11.1	9.32	10.4	10.9
	***N***	**%**	***n***	**%**
Gender				
Woman	105	52.5	33	68.8
Man	93	46.5	15	31.1
Other	2	1.0	0	0.0
Sexual orientation				
Heterosexual	182	91.0	36	76.6
Bisexual	9	4.5	7	14.9
Lesbian/Gay	7	3.5	4	8.5
Other	2	1.0	0	0.0
Relationship status				
Married	102	51.0	26	55.2
Cohabiting	98	49.0	22	46.8
Children				
No	95	47.5	33	70.2
1	40	20.0	2	4.3
2	45	22.5	8	17.0
3	18	9.0	1	2.1
4	2	1.0	1	2.1
5	0	0.0	1	2.1
Ethnicity				
White	184	92.0	41	87.2
Black	5	2.5	1	2.1
Asian	6	3.0	4	8.5
Mixed	2	1.0	1	2.1
Education				
Graduated high school	28	14.0	4	8.5
Some college	38	19.0	4	8.5
Undergraduate	74	37.0	17	36.1
Postgraduate	52	26.0	19	40.4
Other	8	4.0	4	8.5
Employment status				
Employed full‐time	121	60.5	21	44.7
Employed part‐time	23	11.5	6	12.8
Self‐employed	26	13.0	6	12.8
Student	4	2.0	6	12.8
Unemployed	7	3.5	4	8.5
Retired	9	4.5	3	6.4
Employment changed				
No	153	76.5	33	70.2
Yes	47	23.5	14	29.8
Usually work from home				
No	138	69.0	33	70.2
Yes	62	31.0	13	27.7
Country				
UK	119	59.5	32	68.1
USA	17	8.5	4	8.5
Other	64	32.0	12	25.5
Keyworker				
No	166	83.0	44	93.6
Yes	34	17.0	3	6.4
Coronavirus symptoms				
No	179	89.5	39	83.0
Yes	21	10.5	8	17.0

^a^ One interview participant did not complete the baseline and therefore most of the demographic data include data from 47 participants.

### 2.2 Measures

#### 2.2.1 Relational catalyst (RC) support

We measured partner support using a shorter version of the Relational Catalyst Support Survey (Feeney & Collins, 2014). Eight of the original 32 items were included to reduce participant fatigue. These items were selected based on face validity. Because there are no published guidelines on the survey to date, we ran an exploratory factor analysis using principal axis factoring with varimax rotation to examine its factor structure. We found that the scale included two factors: RC support (e.g., “Has given me confidence to pursue my goals or opportunities”; *α* = 0.93) and anti‐RC support (e.g., “Has been negative or demeaning when I am pursuing goals or opportunities”; *α* = 0.86).^3^ The same measures were used in both daily and weekly surveys, but the instructions varied: participants were asked to consider the past 24 hours in the daily measures and the past week in the weekly measures. Participants rated items on a scale from 0 (*Not at All*) to 10 (*Extremely*).

#### 2.2.2 Goal‐related items

At each time‐point, we asked participants to list up to three goals that they had been working toward in the past 24 hours (or the past week in the weekly follow‐ups). Participants reported the following types of goals: domestic (31.4%), exercise/health (20.1%), career (16.4%), hobbies/self‐development (14.7%), relationships (6.3%), self‐care (4.2%), education (2.8%), Covid‐related (2.8%), and finance (1.3%). Participants then answered a set of questions for each goal using one item for each—Goal progress: “How much progress did you actually make toward achieving this goal?”; Goal motivation: “How motivated did you feel in working toward this goal?”; and Goal confidence: “How confident did you feel in being able to achieve this goal?”. Participants were also asked how much they felt the pandemic had affected their goal pursuit overall. The same measures were used in both daily and weekly surveys. However, the instructions varied: Participants were asked to consider the past 24 hours in the daily measures and the past week in the weekly measures. All items were rated on a scale from 0 (*Not at All*) to 10 (*Extremely*), except goal progress which was rated on a scale from 0% to 100%.

#### 2.2.3 Qualitative semi‐structured interviews

We asked participants a range of questions about their relationship and goal pursuit during the pandemic. The questions relevant for this report were: “How have you supported each other during the pandemic in achieving tasks and goals?” and “How has the way in which you support each other changed as a result of the pandemic?”

### 2.3 Fundamental position

The present research was fundamentally guided by pragmatism: the research questions were seen as of primary importance regardless of the philosophical worldview or the method (Creswell & Plano Clark, 2007). While quantitative research is often seen as positivist or postpositivist, these can be at odds with qualitative research, which is inherently more interpretive in nature (Lincoln et al., 2011). Given the unprecedented nature of the pandemic, we believe that using a combination of methods enabled us to gain a more thorough understanding of partner support during the pandemic than using any one method alone could have accomplished.

### 2.4 Quantitative analysis plan

A crucial part of the analysis included separating the within‐ and between‐subjects elements of the predictor variables (see Bolger & Laurenceau, [2013]). The within‐subject variables show the difference in the outcome variables due to within‐person elements and the between‐subjects variable shows the average difference between participants in the outcome variables. Both within‐ and between‐subjects variables were included in the models. Time was scaled to start at 0 and was included in both daily diary and weekly analyses. Daily diary data and the weekly longitudinal data were both separately analyzed using two‐level hierarchical linear modeling (Raudenbush & Bryk, 2002). All participants were measured on the same days, meaning that all variability across days was explainable by between‐participants effects and no additional variance would have been explained by including variability across days. We began each model by including both random intercepts and random slopes for within‐participant variables and time in the models. If the model failed to converge, we removed the random slope of time. However, none of the models converged when including random slopes in the models. Therefore, the final models only included a random intercept. Quantitative data were analyzed using the *lme4* package in *R*.^4^ We used an alpha level of *p* < .01 to account for multiple analyses. Descriptive statistics and zero‐order correlations among variables are presented in Table 2.

**TABLE 2:** Means, standard deviations, and correlations with confidence intervals

Variable	*M*	*SD*	1	2	3	4	5	6
1. RC	6.04	2.76	‐	−0.20** [−0.24, −0.17]	0.08** [.04, 0.12]	0.05 [.01, 0.09]	0.08** [.04, 0.12]	−0.03 [−0.07, 0.01]
2. Anti‐RC	1.07	1.75	−0.14** [−0.17, −0.11]	‐	−0.07** [−0.11, −0.04]	−0.04 [−0.07, 0.00]	−0.01 [−0.05, 0.03]	0.14** [.10, 0.18]
3. Progress	67.13	31.86	0.07** [.03, 0.10]	−0.04 [−0.07, −0.01]	‐	0.52** [.49, 0.55]	0.41** [.37, 0.44]	−0.05 [−0.09, −0.01]
4. Confidence	7.08	2.57	0.08** [.04, 0.11]	−0.00 [−0.03, 0.03]	0.53** [.51, 0.56]	‐	0.60** [.57, −0.62]	−0.01 [−0.05, 0.03]
5. Commitment	7.06	2.64	0.09** [.06, 0.12]	0.03 [.00, 0.07]	0.45** [.43, 0.48]	0.59** [.56, 0.61]	‐	0.02 [−0.02, 0.06]
6. Affect	4.72	3.38	−0.04* [−0.08, −0.01]	0.06** [.03, 0.09]	−0.07** [−0.10, −0.03]	−0.06** [−0.09, −0.03]	−0.03 [−0.06, 0.00]	

Note

*M* and *SD* are used to represent mean and standard deviation, respectively. Values in square brackets indicate the 95% confidence interval for each random measures correlation. The correlations in the daily diary data are presented below the diagonal and weekly measures above the diagonal.

* Indicates *p* < .01.

** Indicates *p* < .001.

### 2.5 Qualitative analysis plan

The qualitative interviews were analyzed using codebook thematic analysis (Braun & Clarke, 2006, 2020) and completed using NVivo 12.0. The authors utilized a combination of inductive and deductive approaches to coding by using previous literature and theory to guide coding but allowing for new codes to be created throughout the coding process. The first and third author coded the interviews; both familiarized themselves with the data before creating the initial low‐level codes. Codes were created by coding each meaning unit, which may have been one word, a sentence, or a paragraph. These codes were then refined iteratively by the two coders and several codes with similar meanings were combined together into themes. Each theme needed to have been mentioned multiple times in order to be included. We also included several subthemes within emotional and instrumental support as these were consistent with the conceptualization of these two types of support in the literature. The final themes were agreed jointly. Any disagreements regarding the classification of codes were discussed until 100% agreement was reached. “[…]” was used in the quotes if unnecessary detail was removed or to provide needed additional information in the quoted data provided. Repeated filler words such as “like” and “yeah” were excluded to aid readability. All identifying information was removed.

## 3 RESULTS

### 3.1 Quantitative results

We expected that perception of RC support would be positively associated with goal outcomes (progress, confidence, commitment) and perception of anti‐RC support would be negatively associated with goal outcomes (H1). The results across daily and weekly analyses were largely consistent (see Table 3).^5^ On days/weeks when a participant perceived their partner as providing RC support, they experienced significantly higher levels of goal progress, confidence, and commitment across goals. Only one of the results was not significant: although consistent with hypotheses and the direction of the other results, including daily goal confidence, participants' perception of RC support was not significantly associated with goal confidence in the weekly analyses. At the between‐participants level, participants who experienced their partners as providing greater RC support overall experienced significantly higher levels of goal progress, confidence, and commitment across daily and weekly analyses compared to participants who experienced their partner as less supportive.

**TABLE 3:** Results from the hierarchical linear modeling for RC and anti‐RC support as predictors of goal outcomes

Predictors	Progress						Confidence						Commitment					
	Daily			Weekly			Daily			Weekly			Daily			Weekly		
	Estimates	CI	*p*	Estimates	CI	*p*	Estimates	CI	*p*	Estimates	CI	*p*	Estimates	CI	*p*	Estimates	CI	*p*
Intercept	65.39	62.66–68.11	**<.001**	66.45	63.76–69.14	**<.001**	7.14	6.93–7.35	**<.001**	7.17	6.95–7.39	**<.001**	7.06	6.84–7.27	**<.001**	7.09	6.86–7.31	**<.001**
RC_W_	1.28	0.64–1.93	**<.001**	1.40	0.55–2.26	**.001**	0.12	0.07–0.17	**<.001**	0.08	0.01–0.15	.028	0.16	0.11–0.21	**<.001**	0.14	0.07–0.22	**<.001**
AntiRC_W_	−0.75	−1.58 to 0.08	.078	−1.73	−2.77 to −0.68	**.001**	0.02	−0.05 to 0.09	.597	−0.04	−0.13 to 0.04	.331	0.10	0.03–0.17	**.005**	0.02	−0.06 to 0.11	.585
RC_B_	1.76	0.74–2.78	**.001**	1.85	0.89–2.81	**<.001**	0.25	0.17 to 0.32	**<.001**	0.25	0.17–0.33	**<.001**	0.27	0.20–0.35	**<.001**	0.27	0.18–0.35	**<.001**
AntiRC_B_	−1.97	−3.70 to −0.24	.026	−2.90	−4.58 to −1.22	**.001**	−0.05	−0.18 to 0.09	.496	−0.13	−0.27 to 0.01	.073	−0.05	−0.18 to 0.09	.490	−0.05	−0.19 to 0.09	.507
Time	0.52	0.09–0.94	.018	0.14	0.03–0.24	**.009**	−0.02	−0.06 to 0.01	.202	−0.01	−0.02 to −0.00	**.003**	−0.01	−0.04 to 0.03	.780	−0.01	−0.02 to 0.00	.070
**Random effects**																		
σ^2^	712.25			664.05			4.72			4.34			4.96			4.67		
τ_00_	264.57_ID_			232.15_ID_			1.49_ID_			1.62_ID_			1.48_ID_			1.65_ID_		
ICC	0.27			0.26			0.24			0.27			0.23			0.26		
*N*	200_ID_			199_ID_			200_ID_			199_ID_			200_ID_			199_ID_		
Observ.	3,755			2,660			3,773			2,676			3,769			2,673		
*R* ^2^	0.036			0.060			0.061			0.081			0.073			0.075		

Bold values indicate the result is significant after Bonferroni correction. Abbreviations: B, between‐participant change; ID, participant as nesting variable; W, within‐participant change.

However, anti‐RC support was less consistently associated with goal outcomes. On the daily level, anti‐RC support only significantly predicted goal commitment. Contrary to our prediction, on days when a participant experienced their partner as providing more (compared to less) anti‐RC support, they experienced significantly more commitment toward their goals. Anti‐RC support did not significantly predict goal progress or confidence at the daily level and none of the between‐participants effects were significant. In the weekly longitudinal analyses, on weeks when a participant experienced their partner as providing more anti‐RC support, they experienced significantly less goal progress. At the between‐participants level, participants who perceived their partner as providing more anti‐RC support made less goal progress compared to participants who perceived their partner as providing less anti‐RC support. Anti‐RC support did not significantly predict confidence or commitment in the weekly longitudinal data.

Furthermore, we also predicted that when participants perceived their partners as providing more RC support, they would perceive the pandemic as affecting their goal outcomes less, and when participants perceived their partners as providing anti‐RC support they would perceive the pandemic as affecting their goal outcomes more (H2; see Table 4). Contrary to the hypothesis, RC support was not associated with the perception that the pandemic affected the participants' goals. However, the results showed that at the weekly level, when participants perceived their partner as providing more anti‐RC support, they were more likely to report that the pandemic was negatively affecting their goal pursuit. The results were not significant during the daily diary. At the between‐participants level, the participants who experienced their partners as providing more anti‐RC support were more likely to report that the pandemic was negatively affecting their goal pursuit compared to participants who experienced their partners as providing less anti‐RC support. The between‐participant results were consistent in the daily and weekly analyses.

**TABLE 4:** Results from the hierarchical linear modeling for RC and anti‐RC support as predictors of perception of goals being affected by pandemic

Predictors	Goals affected by pandemic					
	Daily			Weekly		
	Estimates	CI	*p*	Estimates	CI	*p*
Intercept	5.05	4.66–5.44	**<.001**	4.88	4.49–5.28	**<.001**
RC_W_	−0.06	−0.16 to 0.03	.203	−0.00	−0.12 to 0.12	.982
AntiRC_W_	0.10	−0.02 to 0.23	.112	0.31	0.15–0.46	**<.001**
RC_B_	0.07	−0.07 to 0.22	.308	0.07	−0.07 to 0.21	.339
AntiRC_B_	0.47	0.23–0.72	**<.001**	0.47	0.22–0.72	**<.001**
Time	−0.12	−0.19 to −0.06	**<.001**	−0.01	−0.02 to 0.01	.429
**Random effects**						
σ^2^	6.03			5.12		
τ_00_	4.96_ID_			5.06_ID_		
ICC	0.45			0.50		
*N*	200_ID_			200_ID_		
Observations	1,360			948		
*R* ^2^	0.044			0.047		

Bold values indicate the result is significant after Bonferroni correction. Abbreviations: B, between‐participant change; ID, participant as nesting variable; W, within‐participant change.

### 3.2 Qualitative results

The quotes are accompanied with participant number, gender, and age. In the spirit of qualitative analysis, no frequencies are reported as these would not be meaningful. The themes were organized into five main themes (availability, teamwork, reaching out to others, emotional support, and instrumental support) with emotional and instrumental support themes also including several subthemes within each type of support. Within each theme, there were both positive and negative examples of support. More representative quotes for each subtheme within emotional and instrumental support can be found in Table 5.

**TABLE 5:** Themes and subthemes with descriptions and representative quotes for support

Theme	Subthemes	Description	Quotes
Emotional support	Encouragement & motivation	Support is provided through encouraging partner to start pursuing their own goals and interests	Just motivating talk sometimes just the sentence you know, you can do it. “It's gonna be good. No, you won't fail”. Something like that just lifts my spirit. (#34, M, 18) He helps me a lot with getting motivated and remembering that I have things to do but at the same time, not overworking myself and he encourages me to take breaks and he steps in and just helps me like he'll refill my coffee. (#37, W, 19) When one of us wants to do something the other will just sort of encourage them say “you can do it come on. A little bit more” (#38, M, 33) We do a really good job of motivating each other and keeping each other kind of on track, because we're both in quite a small space together (#41, W, 27)
	Reassurance & validation	Support is provided through reassuring partner so they continue with goal pursuit	Sometimes I have the ability of looking at things in a more rational way. And when he's kind of losing it I try to remind him that we're very privileged in that we are going to be okay. (#2, W, 37) And so we've been supporting each other by: he'll do something and be like, “Oh, I'm not making any progress on this”. I'll say, “but wait, but you did this, this and this”, which are things that I can recognise because I'm outside of it. And then he does the same for me. (#3, W, 26) I think both of us obviously just need reassurance because it is highly anxiety provoking for anyone. So, I think just having to be able to say to the other person, like, “are we going to be alright” for them to just be like, “Yes, I think so”. (#8, W, 27)
	Patience	Support is provided through being considerate and understanding of partner's feelings at times of stress.	I see what he's doing a bit more. Sometimes it's hard to exactly know what his issues are at work on where his stress is really coming from, but now that I see what he's working on, then it makes a little bit easier to understand that. (#14, W, 30) We kind of had these separate work–life, home–life situations. […] I think we just let things go maybe rather than cause an argument about it or kind of a bit more understanding of each other. (#46, W, 31) He is very understanding about food and things like that so he likes to cook when I don't feel like cooking, obviously, because I've had an eating disorder that [is] so special. (#24, W, 23)
	Comfort	Support is provided through affection that is both physical and emotional in times of stress including listening to one's partner and checking in with each other.	If I've got something […] a little bit depressing or something like, just go over. You tell them come and have a short rant or not. And then you also get your cuddles or supporting words. (#10, M, 42) And you see, he makes a lot more space for me to communicate with him than you maybe normally would in that setting, and he kind of listens to it and thinks about it. (#4, W, 46) I think just a lot of checking in with him and talking to him and seeing how he's feeling and what could be helpful. (#5, W, 36)
Instrumental support	Helping with goal	Support is provided through advice and facilitating goal pursuit so that their partner can pursue new goals.	He has been thinking about going back to school, because he didn't finish his bachelor's degree the first time. […] I've been trying to help facilitate him, get into that and see what opportunities might be lurking in the near future when this all ends. (#3, W, 26) Well, she's been quite helpful with my CV and has a look over it and talked about some of the possible options for getting a job after I qualify, which has been helpful. (#31, M, 29) I'll say “do you want to run it by me, and I'll pick up anything before you send it out?” and he seems to like that. (#21, W, 25)
	Taking on other tasks	Completing tasks on behalf of the partner and/or providing financial assistance, to alleviate pressure and allow partner to continue pursuing their own goal.	He financially supports me as well. He always tells me that you don't have to worry about your finances. (#19, M) She's working from home and she seems to think that I'm her personal IT help desk now. So rather than trying to contact anyone, at her actual work, she just bothers and pesters me to fix any IT problems she's got. (#26, M, 40) And he does some of the chores that I absolutely hate, which is nice. And he's just, he's always there, which is nice. He's dependable and he's reliable. (#18, W, 32)
	Non‐interference	Support is provided through physical space so partner can pursue their own goals uninterrupted	I think we're quite supportive of each other's space when we need to, I mean, my partner mostly works in the living room, and I've got the corridor to myself, sometimes it's just a case of closing the door if we need that space when we're working. (#13, M, 31) […] do the things he needs to do and also try and give him a bit of time to do the things he wants to do. […] So, he sits up in his attic and paints these models, so trying to give him time to do that. (#15, W, 36) (NO.14) I think part of this point is just giving him the space for him to play video games and giving me space and just letting each other know like, “okay, are we going to hang out right now? Or are we going to do our own thing for a bit?” (#14, W, 30)

#### 3.2.1 Availability

One of the themes referred to the overall *availability* of partners to support one another during the pandemic. Some participants stated that they were more available to provide each other with support than previously. For example, one participant stated that “It's nice to be helpful. I'm still having a break from work. And I see what he's doing a bit more. Sometimes it's hard to know exactly what his issues are at work or where his stress is really coming from, but now that I see what he's working on, it makes it a little bit easier to understand that” (#14, W, 30). However, other participants said that their partner had not been available for support since the pandemic started: “I'd say at this point he's not really available emotionally to support me and also what I would need support in is keeping me motivated to apply for jobs and that's not really going on at the moment. He's really stressed. He's running around, running against a lot of deadlines and personal work like for his dissertation and other endeavours” (#12, W, 26). Another participant said their partner had gotten a bit better but was still not very good at providing support during this time: “He gives me time to do stuff and he's getting a bit better at this but like my couch to five k, I need to go and he has been a bit rubbish [if] I want to go out at say 11 o'clock and he might fall off and not come down to like midday while I'm starving” (#15, W, 36).

#### 3.2.2 Teamwork

Participants also spoke about the need for *teamwork* and flexibility during the pandemic due to the changes in current circumstances. Some participants said they were struggling to cope with the pandemic but tried to ensure they would talk to each other to help support one another: “At least we don't have to worry about the kids but it has been really crazy time. We constantly say that it just doesn't feel real, that you wouldn't have thought that something like this can happen within our lifetime. […] We sort of convince each other to stick to the lockdown, because that's the best thing you can do to minimize the risk” (#29, W, 32). Another participant remarked that the situation was not ideal but they were making pragmatic decisions to be able to work together: “I work upstairs in a small room whereas he has the whole of the downstairs because his job just needs a bigger computer setup. He's on the phone a lot and he needs space for all his stuff as I can work from the small computer and a little desk, which isn't ideal. I'm used to being in a big open office. I feel a bit confined. But I can work like this, which makes my life easier if he carries on like that” (#46, W, 31). Furthermore, some participants spoke about changing things around to support one another and avoid boredom: “I would usually do the brunt of the housework and cooking, and he's taken a lot of that off my hands and then I've gone outside and I've done more gardening, which would have usually been his job” (#33, W, 29).

#### 3.2.3 Reaching out to others

Sometimes participants felt that they needed to reach out to other people outside of the relationship for support or to encourage their partner to do so if they felt the partner was struggling. For example, one participant stated that: “[I'm] encouraging him to keep in touch with his parents and his friends and doing all those sorts of things because he's a bit of hermit sometimes. And I think given half a chance he would just not have talked to them if he didn't have to, but he will miss them and want to see them but at the same time, he'll sometimes forget that he needs to pick up the phone. So, I try to make time to do that with him and go ‘Let's give him a ring’” (#15, W, 36). Another participant said she had asked for financial support from parents primarily to help her partner: “I did speak to my parents. This was mainly to support him to be fair about borrowing a bit of money to tide us through because obviously we don't know when wages are coming in and everything” (#8, W, 27).

#### 3.2.4 Emotional support

Participants reported providing each other at least some form of emotional support. They identified two subthemes that were directly related to goal pursuit: *encouragement and motivation* and *reassurance and validation*. Participants identified *encouragement and motivation* as a form of support that they or their partners provided to help motivate each other to pursue goals and interests. For example, one participant noted their partner was encouraging them to pursue goals outside of work: “[He] encouraged me to take the time to pursue a goal, like with the foreign language. And he said, ‘you know, I'm going to work on a course now. Pick up yours, take an hour and just do something different’. So, we're quite encouraging of each other to not just be enveloped in work and to pursue passion projects instead” (#44, W, 30). Alternatively, some participants were unwilling to provide encouragement or motivation when they felt it was their partner's decision. For example, one participant stated, “I never want to seem pushy. I'm more likely to stay quiet unless I have a strong opinion on something” (#21, W, 25).

*Reassurance and validation* were also identified as aiding goal pursuit by many participants. While encouragement and motivation related to the initial pursuit of goals, reassurance and validation were identified as aiding the continuation of goal pursuit. Participants noted that reassuring words were needed to support them throughout their tasks so that they continued to feel capable. One participant noted their partner was supporting them through validating their daily achievements and encouraging forgiveness: “I think I'm making sure he forgives himself when he's not super‐productive. He wakes up and he's like, ‘if I work eight hours today, it'll be a good day’. So, I tell him ‘and even if you only work five hours, it was a good day. It wasn't a great day, but it was a good day.’” (#12, W, 26). On the other hand, some participants noted it was not always appropriate to comment on or encourage certain behaviors as “the other person kind of takes offence to it” (#36, W, 52). One participant noted they were able neither to encourage nor to reassure their partner during the pandemic as they struggled to get out of a negative mindset and hence had to “minimize contact” (#37, W, 19) to not negatively affect their partner.

The other emotional support subthemes did not directly relate to goal pursuit but rather how partners communicated with one another: *patience* and *comfort*. Some participants noted an increased level of *patience*. Participants identified patience as being utilized in times of stress to understand and be considerate of their partners' feelings. For example, one participant said “He's probably got a lot more patience in listening to me rambling on about things where normally he would just be like ‘this is a total non‐event, what are you doing?’” (#4, W, 46). Many participants also noted an increase of physical and emotional *comfort*. For example, one participant said “we kiss and we hug a lot, and that's a way in which we like to show each other support” (#24, W, 23). Nonetheless, some participants noted they were less patient with one another and were often “slipping into sort of petty disagreements […] [that were] little and often” (#44, W, 30). The conflicts discussed by participants were often not goal specific but a general frustration that extended into their relationship.

#### 3.2.5 Instrumental support

Instrumental support was also reported during lockdown. Instrumental support ranged from actually helping with the goal itself, to helping with other tasks to take a load away from each other, and finally not interfering and instead giving each other the space and time to pursue goals individually. *Helping with a goal* was reported by participants as one of the ways to provide instrumental support. Participants noted that tangible and informational help including giving advice was an important factor in providing support for starting new goals as well as continuing with existing goals. For example, one participant said this about their partner: “She's helping me look for adventures like books, like part‐time work, even volunteer work to see if we can help at the hospitals” (#26, M, 40). However, some participants noted that at times they felt unable to help their partner as they needed to focus on their own tasks. For example, one participant stated, “it's harder to define those boundaries between work and home life” (#3, W, 26). As such, it was at times difficult to balance assisting their partner alongside pursuing their own goals.

Participants also reported that they had each been *taking on other tasks* to aid one partner's goal pursuit. Participants noted that they increased support on a variety of tasks including household, childcare, and financial assistance instead of being directly involved in helping a partner to pursue their goals. As such, some participants also noted they were taking turns on managing children or household responsibilities to allow the other to pursue goals. Some female participants noted that gender dynamics played a role within household and childcare responsibilities as they described themselves as housewives. For example, “I feel a bit like a 1950s housewife at the moment” (#15, W, 36). Overall, some participants said that both partners had become more flexible in taking on chores when one partner needed help. For example, one participant said “when I was trying to learn a language, he would make sure he took the kids and then I had some time just to focus on it myself” (#33, W, 29).

Finally, participants reported that giving each other time and space to pursue goals was a necessary form of support during the pandemic (*non‐interference*). Within this theme, participants did not take on additional tasks themselves to give the other space but rather would stay out of each other's way when they knew one partner needed to concentrate on their goals. For example, one participant said “when we did work, we worked in separate rooms. […] So, when one has something to do, it's not interfering. No chat, or nothing” (#20, W, 29). This was not always possible for participants, with some noting that due to space, “it is too difficult to separate the work and not work” (#22, M, 47).

#### 3.2.6 Follow‐up interviews

The follow‐up interviews a month later reflected much of the same themes as in the original interviews, with participants largely reporting no change. This suggests that participants were still behaving in the same ways after having been in lockdown with each other for over a month. Some participants mentioned an increase in comfort and affection: “There's definitely been more hugs” (#11, W, 36). Participants also stated that there was increase in teamwork, which typically presented as taking on other tasks and “taking it in turns to do things” (#15, W, 36). Additionally, a theme of increased quality time was mentioned by participants. For some participants, this was presented as ensuring they always spent the evenings together whereas others would schedule in date nights: “like date nights basically even though it's a date night watching a film in our own house” (#15, W, 36). Overall, the themes identified suggest participants felt an increased sense of togetherness as the lockdown continued.

### 3.3 Mixed methods results

The mixed methods approach allows for comparison between the quantitative and qualitative results and can be complementary. The results showed that the survey participants rated overall level of RC support relatively high and anti‐RC support relatively low during the pandemic. In the qualitative interviews, some participants reported that they were unable to provide support to each other during the pandemic but the incidence of anti‐RC support was rare. The qualitative findings also provide further nuance into the types of support: participants reported both emotional and practical support, which were further divided into support that was directly relevant to goal pursuit and support that was enabling support indirectly. We did not find evidence of a distinction between emotional and practical support in the exploratory factor analysis of the quantitative survey. Instead, only positive and negative RC support were identified. This may, however, reflect that participants find that their partners provide both types of support equally and there may be an opportunity in the full RC support scale to better distinguish between emotional and practical support.

## 4 DISCUSSION

The present study provides a unique perspective into how individuals in relationships are coping during one of the worst global public health crises the world has ever experienced. The current pandemic is an unprecedented and stressful event that has an unclear ending and is surrounded with uncertainty and change. Partner support during this time is especially crucial given that support from outside sources may not be easily accessible. Previous research on partner support and goal outcomes has mostly been conducted during non‐stressful times (Feeney et al., 2017; Jakubiak & Feeney, 2016; Kumashiro et al., 2007; Overall & Fletcher, 2010) or when only one member of the couple was experiencing the stressor (Bolger et al., 2000; Crockett et al., 2017; Gleason et al., 2008). Furthermore, the thriving through relationships framework suggests that RC support is important in non‐stressful situations only. However, we showed that RC support can still be beneficial in a stressful situation, at least for goal outcomes: we found that RC support was associated with better goal outcomes (progress, confidence, commitment) during the pandemic. Anti‐RC support was less robustly associated with goal outcomes but participants who reported their partners as providing more anti‐RC support overall were much more likely to perceive that the pandemic was affecting their goal pursuit. It may be that some participants perceive that the pandemic is affecting their goal pursuit because their partner is more interfering and getting in the way of goal pursuit.

In addition to replicating the previous findings on partner support in a situation in which both partners were experiencing a stressor simultaneously, the study also provides the first evidence that self‐reported perception of RC support is predictive of thriving outcomes (Feeney & Collins, 2015). Many of the themes found in the qualitative analyses also support the theoretical conceptualization of RC support: effective support includes being available to each other when needed and providing both emotional and practical forms of support; support is important throughout the goal pursuit process from helping recognize opportunities to celebrating successes; and support may also involve helping the support recipient recognize and find resources (e.g., enlisting others) to help them achieve their goals. Together, both the quantitative and qualitative findings provide further support for the theory of thriving through relationships.

The qualitative results also provided further insights into the types of support provided. Previous research has examined the role of emotional and instrumental support on a variety of outcomes with results generally being mixed (Jakubiak et al., 2019; Morelli et al., 2015; Shrout et al., 2006). Some researchers have shown that visibility of support can explain why in some instances support is beneficial but not in others (Girme et al., 2013; Jakubiak et al., 2019; Zee & Bolger, 2019). These findings generally suggest that support that is not perceived by the recipient is beneficial whereas there can be costs to perceived support. However, many other studies do show that perceived support is associated with greater individual and relational outcomes (Fitzsimons & Finkel, 2010; Jakubiak & Feeney, 2016; Rusbult et al., 2009). The findings from our qualitative results may shed some light on this debate: participants identified both directly goal‐related support (e.g., providing encouragement and motivation, helping with the goal) as well as support that was only indirectly linked to goals (e.g., providing comfort, helping with other tasks). Indeed, this type of indirect RC support may be particularly important during the pandemic due to increased childcare and household responsibilities; partners who are able to share these responsibilities are likely to be able to make more progress toward their goals whereas having a partner who is unable to provide support indirectly may hinder goal pursuit. It is possible that existing measures on support do not capture well indirect forms of support which may not be perceived as support by the recipient but is labelled as support by the provider. Indirect forms of support can be especially helpful in enabling the recipient to make progress toward their goals without negatively impacting self‐efficacy. Future research should investigate these findings further in quantitative studies.

The study provided a unique perspective into how individuals in relationships are supporting each other in order to manage goal pursuit during a global pandemic. While the results were collected during the pandemic, and some questions were specific to the pandemic (e.g., how much goal pursuit had been affected by the pandemic), we expect these findings to generalize beyond the current situation. For example, RC support is not unique to the pandemic and while some interview participants reported that the support had changed since the beginning of social distancing measures, the multiple ways in which people provide support are likely to be similar both in and out of the pandemic.

The study also has several practical implications. The Gottman Method (Gottman & Schwartz Gottman, 2008), a model of couple's therapy, includes “making life's dreams come true” as important for relationships. It refers to having discussions about how the relationship can help achieve individual goals. Discussing goals in the presence of one's partner and asking for what one needs from the partner to achieve their goals should be an important element of a couple's therapy. Furthermore, it may be important to provide psychoeducation to couples on how to cope with the stressful situation in order for each partner to continue to pursue goals during the pandemic. For example, interventions based on dyadic coping research provide psychoeducation on how stress can affect couple functioning. A 3‐phase training is also conducted as part of the interventions to enhance dyadic coping and mutual understanding of functioning of each partner (Bodenmann & Randall, 2012). The qualitative results can be also used to provide strategies to the public on how to provide effective support during the pandemic. For example, it is important to help partners recognize opportunities, build up their confidence, and be emotionally available in case of setbacks.

Additionally, the interview participants spoke about giving each other space to pursue goals when needed. This was considered an important form of indirect support and is consistent with attachment theory's notion of providing a secure base for exploration in which a partner is only interfering when absolutely necessary (Feeney & Thrush, 2010). These results are also consistent with a recent qualitative study into dyadic coping and self‐regulation in homes for a chronically ill person (Sallay et al., 2019). The study found that space use was an important element of dyadic coping and not managing the space well caused conflicts akin to the present study. Therefore, these findings suggest that it may be important for therapists to consider how couples manage space around each other both when partners are wanting to pursue goals or when they are coping with distress and needing space to be alone.

### 4.1 Strengths and limitations

The present study had several strengths. These include the use of mixed methods which benefit from the generalizability and reproducibility of statistical analyses as well as an in‐depth account of participants' experiences. Additionally, longitudinal data was obtained with daily and weekly reports recorded alongside multiple measures of each construct over the first weeks of lockdown rather than relying on a single observation. Nonetheless, there are several limitations that should be considered. The data were collected from individual couple members, not dyads. As such, reports regarding their partner's behavior may not be as accurate as the reporting of their own behavior. Therefore, it was not possible to assess questions such as support visibility.

It is also possible that the study only captured participants who are functioning well during lockdown given that participants across quantitative and qualitative data reported relatively high levels of support. Some anecdotal evidence from China suggests that the pandemic is likely to “make or break” relationships (Liu, 2020). The present study may be better able to speak to how to cope well and less about what may cause couples to break during the pandemic. Furthermore, during the COVID‐19 pandemic, both partners in a couple are simultaneously experiencing the same stressor. In non‐pandemic times, stressors may often fall only on one individual (e.g., preparing for exams, work stress, illness of a parent) and the stressor may only be indirectly affecting the non‐stressed partner (Falconier & Kuhn, 2019). Therefore, although consistent with previous literature, these findings may only generalize in situations in which both couple members experience the same stressor simultaneously (e.g., having a sick child, political unrest, recession, or dealing with natural disasters such as earthquakes, hurricanes, fires, or floods). Moreover, because of the rapid development of the pandemic, it was not possible to collect pre‐pandemic data and examine how support or goal outcomes have changed quantitatively; rather our results rely on the qualitative participants' retrospective accounts of their pre‐pandemic levels of support to assess support change.

Participants' stress level was not explicitly measured in the present study and therefore it is not clear how much stress participants were experiencing due to the pandemic. Future research on goal pursuit during the pandemic would benefit from an explicit measure of stress. The age of children in the home may influence partners' ability to pursue goals. This was, however, not measured in the present study. Therefore, it is not clear how big a role children played in partners' ability to pursue goals during the pandemic. The random slopes in the models failed to converge, which resulted in an inability to estimate different slopes for different individuals. This may be because we did not have sufficient variance in the data to estimate random slopes and future research with a greater number of time‐points may enable researchers to estimate the random slopes in addition to random intercepts. Additionally, while we used longitudinal data in the study, the analysis does not involve any results on change over time due to different goals being assessed each day.

### 4.2 Future directions

There are a number of possible directions for future research. The qualitative interviews highlighted that support can be both direct and indirect and the participants discussed a number of direct and indirect forms of emotional and instrumental support. We are aware of no previous studies that have explicitly examined whether direct and indirect forms of support are differentially associated with goal outcomes. Therefore, it would be especially interesting in future research to examine whether recipients recognize indirect forms of support as support, and whether indirect support may account for the mixed findings across the support literature in addition to support visibility. Furthermore, because the present sample consisted primarily of individuals in relationships who were coping relatively well during the pandemic, future studies should aim to capture more partners who are not coping well (e.g., those in couples therapy) to better understand both extremes. We also acknowledge that having other family members in the household (e.g., children) may also be affecting goal pursuit during the pandemic, but this was not explicitly addressed in the present study. Future research would benefit from explicitly examining how having children may have affected support for goal pursuit during the pandemic.

Moreover, we measured support toward goals in general rather than support for specific goals. However, it is likely that different goals require different forms and amount of support. Indeed, providing support that is consistent with the needs of the recipient is a part of being a skilled support provider (Rafaeli & Gleason, 2009) and the interview participants discussed a number of ways in which they and their partner provided support for each other's goal pursuit and how this support varied depending on need. Therefore, it would be interesting to examine in future research whether different goal types (e.g., health, career, relationship) would require different types of support and whether this would have an impact on goal outcomes. Furthermore, it is possible that high goal confidence means high self‐reliance for some participants. The results from the present study showed that RC support was positively associated with goal confidence, suggesting that this was not the case overall. However, future research could further disentangle goal confidence from self‐reliance.

Finally, the focus of the present study was primarily on how partners have provided each other with support toward goals during the pandemic and whether this support had changed as a result of the pandemic. Therefore, we did not explicitly focus on how support emerges in relationships or how providing effective support may catalyze relationships over time. For example, given the lack of research into the role of the support seeker in seeking support in relationships (Feeney & Collins, 2015), future research should focus on the ways in which support seekers can elicit support from their partner. Furthermore, previous quantitative research has established that partner support is beneficial for relationship outcomes (Overall et al., 2010). However, it would be interesting to further examine the ways in which support can improve relationship outcomes (e.g., by developing greater connection between partners, increasing appreciation) in qualitative studies.

## 5 CONCLUSION

In conclusion, this mixed methods study provided both quantitative and qualitative evidence of how partner support and goal outcomes are impacted during COVID‐19. The quantitative findings show that perception of greater partner RC support is positively associated with better goal outcomes. Qualitative findings highlight the importance of both direct and indirect forms of partner emotional and instrumental support to enable goal pursuit. The study adds to the present literature by showing that RC support can still be beneficial in stressful times alongside source of strength support, and shows that both direct and indirect forms of support may be needed in order to make progress toward tasks and goals. Most participants in the study exhibited an amazing amount of resilience in the face of the pandemic, with many of the participants reporting increased support. This suggests that individuals who are in supportive relationships may be able to grow individually and in their relationship by experiencing adversities together.

## CONFLICT OF INTEREST

There are no conflicts of interest to disclose.

## ETHICAL APPROVAL

The study received ethical approval from the authors' institutional review board and all participants consented to participate in the study.

## TRANSPARENCY STATEMENT

Data, code, and materials can be found at https://osf.io/qr7cm/?view_only=365bf35f7ddd45548143b851e10cfcd9.

## Data Availability

All data, code, and materials along with the preregistration are available on the Open Science Framework with anonymized links in the manuscript.

## Footnotes

^1^
We did not invite Prolific participants to participate in the follow‐up interviews because the quantitative study period had ended. The four other participants who were not invited for the second interview completed their first interview around the time the first second interviews took place and therefore the study period ended before they would have been invited to the second interview.

^2^
Individuals working in critical roles such as healthcare during the pandemic.

^3^
We were unable to estimate reliability change (Bolger & Laurenceau, 2013) because the models to calculate the estimates did not converge. Therefore, we provide a more traditional Cronbach's alpha as an estimate of reliability.

^4^
We did not perform any lagged analyses because participants reported on support toward up to three specific daily/weekly goals/tasks that may have been very different on different days/weeks. Therefore, the support required one day may be very different from support required the next day. For example, a participant may have had a work deadline the day before and needed their partner to provide support by looking after the children more. The next day their goal may have been to spend more time with the children and thus they did not need support from their partner.

^5^
We also tested models including COVID‐related covariates (change in employment, keyworker, COVID‐symptoms, working from home, days since social distancing) . Very few were significant or changed the results and can be found as part of the code/results on OSF.
